# Influence of Polycyclic Aromatic Compounds and Oxidation States of Soot Organics on the Metabolome of Human-Lung Cells (A549): Implications for Vehicle Fuel Selection

**DOI:** 10.1021/acs.est.3c05228

**Published:** 2023-11-13

**Authors:** Lina Wang, Wen Wen, Jiaqian Yan, Runqi Zhang, Chunlin Li, Hongxing Jiang, Shaofeng Chen, Michal Pardo, Ke Zhu, Boyue Jia, Wei Zhang, Zhe Bai, Longbo Shi, Yingjun Cheng, Yinon Rudich, Lidia Morawska, Jianmin Chen

**Affiliations:** † Shanghai Key Laboratory of Atmospheric Particle Pollution and Prevention (LAP^3^), Department of Environmental Science and Engineering, Fudan University, Shanghai 200438, China; ‡ Shanghai Institute of Pollution Control and Ecological Security, Shanghai 200092, China; § Department of Earth and Planetary Sciences, Weizmann Institute of Science, Rehovot 76100, Israel; ∥ School of Ecology and Environment, Inner Mongolia University, Hohhot 010021, China; ⊥ International Laboratory for Air Quality and Health (ILAQH), School of Earth of Atmospheric Sciences, Queensland University of Technology, Brisbane, Queensland 4001, Australia; # IRDR International Center of Excellence on Risk Interconnectivity and Governance on Weather/Climate Extremes Impact and Public Health, Institute of Atmospheric Sciences, Fudan University, Shanghai 200438, China

**Keywords:** combustion soot, oxidation state, lung cells, metabonomics, oxygen-containing PAH, biodiesel

## Abstract

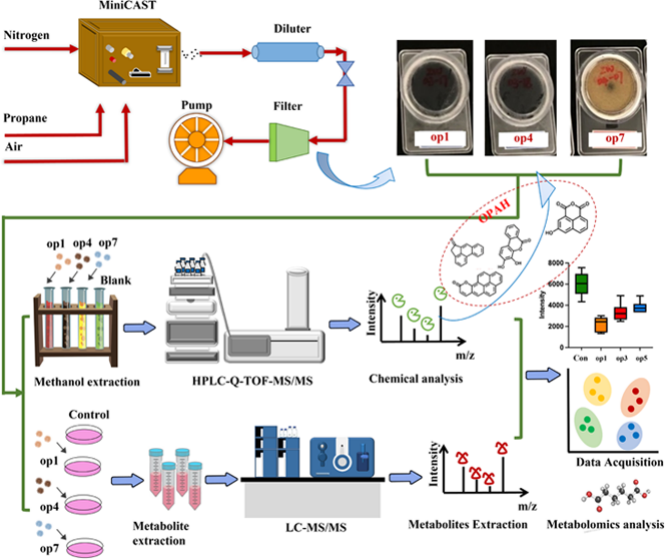

Decades of research have established the toxicity of soot particles resulting from incomplete combustion. However, the unique chemical compounds responsible for adverse health effects have remained uncertain. This study utilized mass spectrometry to analyze the chemical composition of extracted soot organics at three oxidation states, aiming to establish quantitative relationships between potentially toxic chemicals and their impact on human alveolar basal epithelial cells (A549) through metabolomics-based evaluations. Targeted analysis using MS/MS indicated that particles with a medium oxidation state contained the highest total abundance of compounds, particularly oxygen-containing polycyclic aromatic hydrocarbons (OPAHs) composed of fused benzene rings and unsaturated carbonyls, which may cause oxidative stress, characterized by the upregulation of three specific metabolites. Further investigation focused on three specific OPAH standards: 1,4-naphthoquinone, 9-fluorenone, and anthranone. Pathway analysis indicated that exposure to these compounds affected transcriptional functions, the tricarboxylic acid cycle, cell proliferation, and the oxidative stress response. Biodiesel combustion emissions had higher concentrations of PAHs, OPAHs, and nitrogen-containing PAHs (NPAHs) compared with other fuels. Quinones and 9,10-anthraquinone were identified as the dominant compounds within the OPAH category. This knowledge enhances our understanding of the compounds contributing to adverse health effects observed in epidemiological studies and highlights the role of aerosol composition in toxicity.

## Abstract 2

This study concluded that OP4 generated more OPAH by liquid chromatography-mass spectrometry (LC-MS) and MS/MS technologies, which may cause oxidative stress and metabolic damage.

## 1 Introduction

The World Health Organization (WHO) and the Global Burden of Disease (GBD) have recognized the significant risk that soot particles pose to human well-being, leading to increased morbidity and mortality rates.^1^ According to the WHO, approximately 7 million people worldwide die prematurely annually due to exposure to both outdoor and indoor air pollution, with 4.3 million deaths attributed to the indoor combustion of solid fuels.^2^ The combustion process generates a substantial quantity of soot particles, which harm the environment and human health.^3^ Although there is a lack of standardized methods for assessing the economic costs associated with mortality and morbidity from exposure to soot particles from combustion, available data demonstrate that reducing pollution from burning can yield substantial economic benefits.^4−6^

Incomplete combustion results in the formation of soot particles predominantly composed of black carbon, accompanied by minor carbonaceous and inorganic constituents, which are closely intertwined with particle formation, growth, and aging processes.^7−9^ The combustion conditions, including fuel type, combustion state, and oxygen availability, significantly impact the production and properties of soot particles.^10,11^ Numerous studies have examined the physicochemical properties and health implications of black carbon and organic compounds present in fresh and aged soot particles.^12−15^ However, there is currently no consensus regarding the toxicity of soot and the effects of different oxidation states and critical compounds that contribute to particle toxicity.^16^

Previous studies have provided evidence indicating that soot particles resulting from biomass burning are linked to an increased risk of acute and chronic lower respiratory infections, which can lead to lung inflammation, fibrosis, and chronic obstructive pulmonary disease (COPD).^17−20^ Exposure to particles emitted from coal combustion has also been associated with potential lung damage.^18,21^ Moreover, emissions from diesel vehicles have been found to downregulate genes in mitochondrial complexes I and V, contributing to COPD and disruptions in carbohydrate,^19^ nucleotide, cofactor, and vitamin metabolism.^20^ Furthermore, studies have reported that black carbon within combustion particles can induce oxidative stress and trigger an inflammatory response in both in vivo and in vitro experiments.^22^ Researchers suggest that polycyclic aromatic hydrocarbons (PAHs) and associated organic compounds are the most toxic components present in organic aerosols derived from biomass burning.^23^ The primary toxicological mechanism associated with exposure to atmospheric particles is believed to be the induction of oxidative stress, which arises from an imbalance between oxidants (reactive oxygen species, ROS) and antioxidants. Combustion processes encompass a range of fuel–air ratios and undergo various aging processes, resulting in the formation of particles with diverse oxidation states and chemical compositions that can give rise to a wide range of health effects. Given the diverse health impacts and complex chemical composition of soot particles, it is crucial to gain a comprehensive molecular-level understanding to uncover the underlying causes of their toxicity.^24^

MiniCAST generators, which utilize propane as a fuel source, are commonly employed in laboratory settings to generate combustion particles. Ess et al. concluded that the physicochemical properties of soot particles generated by the miniCAST closely resemble those produced by practical engines.^25^ These generators offer convenience in manipulating combustion conditions, such as fuel type and airflow, thereby enabling the production of soot particles with varying oxidation states and diverse chemical compositions. For example, Malmborg et al. observed that higher temperatures promoted the growth of conjugated polycyclic aromatic hydrocarbons (PAHs) generated by a miniCAST generator.^26^ Le and Ess et al. employed Raman spectroscopy to examine the organic components produced by miniCAST combustion and identified particles containing significant carbonyl compounds under fuel-rich conditions.^25,27^ The operational parameters of the miniCAST device influence the physicochemical properties of the resulting particles.^28−30^ Ess et al. concluded that the physicochemical properties of soot particles generated by the miniCAST closely resemble those produced by practical engines.^25^ Mamakos et al. conducted an investigation into particle sizes under various oxidation states; the results of their inquiry revealed that particles generated across different oxidation states all fell within the category of ultrafine particles (Dp < 100 nm).^31^

Motivated by proving that soot particles generated from different combustion conditions or fuel types exhibit varying toxicity levels and health effects due to differences in their chemical composition and oxidative properties, this study employed untargeted mass spectrometry to assess the physicochemical properties of soot particles generated under various oxidation states, identifying critical compounds contributing to metabolomic changes on human alveolar basal epithelial cells (A549) (specifically, OPAHs), elucidating molecular-level mechanisms of oxidative stress induction, and assessing the real-world emissions.

## 2 Materials and Methods

### 2.1 Soot Particle Preparation

A particle combustion standard burner (Model 5202 miniCAST Jing Ltd. Switzerland) was employed to generate soot particles. The combustion device operates by extinguishing the diffusion flame of a propane airflow at a fixed height through the introduction of N_2_, resulting in a flow containing soot particles (Figure S1). By adjusting the flow rates of propane, air, and nitrogen and manipulating the fuel-to-air ratio in the flame, we obtain particles with diverse oxidation states, compositions, size distributions, and structures. A charcoal denuder was utilized to eliminate the gaseous emissions. For this study, three distinct combustion conditions were chosen to generate particles representing different oxidation states (achieved through varying fuel-to-air ratios), each characterized by unique chemical compositions, molecular features, and cytotoxicity. Figure S1 illustrates the miniCAST sampling setup for the three oxidation conditions. For an increased subscript digit of op_i_, an increased N_2_ flow and reduced oxidation airflow with the fuel gas were adopted to lower the adiabatic flame temperature (op means oxidation potential; op1: high oxidation potential; op4, medium oxidation potential; op7: low oxidation potential). Emissions at each op state were sampled onto quartz fiber filters (47 mm, Whatman, QM-A) for 20 min at a flow rate of 20 L/min. The sampling was repeated at least six times for each op state. The collected samples were stored at −20 °C for future processing and analysis.

### 2.2 Sampling of Vehicle Emissions Using Various Fuels

This study collected exhaust emissions from two Jiefangyi open van trucks, which comply with the National Emission Standard GB17691-2005 (China V), running on diesel and biodiesel fuels, as well as exhaust emissions from a gasoline-fueled midsize MPV (92#) and a natural gas truck (model: SX4184ZL301TL) (Supporting Information 2.2 S). The main technical parameters are shown in Supporting Information Figure S2 and Tables S1–S3. The fuels used were B5 biodiesel and diesel fuel (0#). The emissions were collected onto a quartz-filter cartridge (Size: 28 × 70 mm, Qty: 25/PK, Whatman Thimble Silica) using an automatic PM_2.5_ sampler (3012H, Qingdao Laoying Co., China) operating at a high flow rate of 30 L/min, lasting for 30 min.

### 2.3 Chemical Analysis of Organics Coating on Soot Particles with Three Oxidation States Using High-Resolution Mass Spectrometry

The compounds extracted from the soot particles of op1, op4, and op7 were analyzed using an HPLC-Q-TOF-MS system equipped with a C18 column (SB-C18, 3.0 × 100 mm, 1.8 μm, Agilent Technologies). The system consisted of high-performance liquid chromatography (HPLC, 1290 Series, Agilent Technologies) and a G6546 series quadrupole-time-of-flight mass spectrometer (Q-TOF-MS) for the separation and analysis of compounds based on polarity, thermal stability, refractory gasification, and the presence of macromolecules. First-order and second-order mass spectra were acquired using MS and targeted MS/MS modes, respectively, to identify and analyze the structures of unknown substances.

For ionization of the neutral compounds, both positive (ESI+) and negative (ESI−) electrospray ion sources (ESI) coupled to time-of-flight mass spectrometry (TOF-MS) were employed. The C18 column (SB-C18, 3.0 × 100 mm, 1.8 μm, Agilent Technologies) operated at a flow rate of 0.4 mL/min with a sample volume of 2 μL. The mobile phases consisted of water (A) and methanol (B), both containing 0.1% formic acid. The mass scan range was set from *m*/*z* 50 to 1200 Da, and helium was used as the carrier gas. The dry airflow was maintained at a flow rate of 7 L/min, while the sheath air temperature and flow rate were set at 350 °C and 11 L/min, respectively.

The filter samples collected for both MiniCAST and vehicle emissions were cut into pieces and placed in a 50 mL conical flask. Then, 20 mL of methanol was added, and the flask was sealed for 30 min of ultrasonic treatment. The methanol extract was filtered by using 0.22 μm poly(tetrafluoroethylene) (PTFE) filters to remove insoluble components. The filtrate obtained by a 0.22 μm membrane filter was concentrated to 1–2 mL using a nitrogen flow in a water bath at 55 °C and then transferred into a 1 mL vial. Methanol extracts of organic components for the selected samples were analyzed by using the HPLC-Q-TOF-MS analysis. Blank membrane samples underwent the same treatment. Prior to sample analysis, instrument calibration was conducted using a diluted standard tuner with ionic mass charge ratios of 112.98558 and 1033.9881 Da. MS and targeted MS/MS modes were employed to identify the chemical structures of the compounds. The unsaturated properties of organic compounds were determined based on their double bond equivalent (DBE) (Supporting Information 2.3 S) and aromatic equivalent (*X*_c_) values. *X*_c_ is calculated according to eq 1
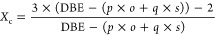
1in which *p* and *q* correspond to the ratio of oxygen and sulfur atoms involved in the π bond structure in the compound, depending on the category of the compound. For compounds detected at positive and negative modes, the values of *p* and *q* are assumed to be 0.5. Values of *X*_c_ ≤ 2.5000, 2.5000 ≤ *X*_c_ ≤ 2.7143, 2.7143 ≤ *X*_c_ ≤ 2.8000, 2.8000 ≤ *X*_c_ ≤ 2.8330, and 2.8330 ≤ *X*_c_ ≤ 2.9230 represent the molecular structure containing no ring, single ring, naphthalene, and pyrene, respectively.

### 2.4 Quantification of PAHs, OPAHs, and NPAHs in Vehicle Emissions Using Various Fuels

Prior to injection, the filter membrane samples were cut into pieces and subjected to ultrasonic treatment, filtration, and extraction with dichloromethane. The filtrate was then evaporated, and the volume was adjusted to 1 mL with dichloromethane. Hexamethylbenzene was added as an internal standard to achieve a concentration of 100 ppb. The blank membrane samples were processed by following the same procedure as described above. The samples were analyzed by using gas chromatography–mass spectrometry (GC–MS) (THERMO TRACE 1300/ISQ7000). The chromatographic column used was an HP-5 ms quartz capillary column (30 m × 0.25 mm × 0.25 μm). The GC–MS system operated in splitless injection mode with an injection volume of 1 μL and a column flow rate of 1.2 mL/min. The temperature program was as follows: initial temperature of 60 °C held for 4 min, followed by a ramp of 8 °C/min to 220 °C and held for 2 min, and finally a ramp of 6 °C/min to 310 °C with a hold time of 15 min. The entire experimental process adhered to strict quality control (QC) measures, including the insertion of a standard sample to monitor instrument performance after every 10 samples.

### 2.5 Analysis of Organic Carbon

The organic carbon content in samples of vehicle emissions using various fuels was measured using a thermal/optical analyzer (DRI Model 2000) based on the thermal/optical reflectance method specified by IMPROVE. The filter samples were placed in sample boats and subjected to continuous heating in a pure helium gas atmosphere at temperatures of 140, 280, 480, and 580 °C to determine the concentrations of OC1, OC2, OC3, and OC4, respectively. Subsequently, in a 98% helium and 2% oxygen gas atmosphere, the carbonaceous gases were catalytically oxidized to CO_2_ and then reduced back to methane for detection. Prior to testing, the instrument underwent sample furnace leak testing, a 30 min peak check (to ensure system stability), and a 30 min run blank (without samples). The accuracy of the instrument was validated using methane of known mass, and calibration was performed using a sucrose solution. The final OC concentration was calculated as OC1 + OC2 + OC3 + OC4 + OP.

### 2.6 Cell Exposure by Soluble Soot Extracts of Different Oxidation States and Metabolic Analysis

After complete drying, the methanol extracts obtained from the three combustion conditions were reconstituted in Milli-Q water to obtain specified concentrations before exposing the cells to further treatment. Subsequently, cultured A549^32^ were exposed to the extracts of the soot particles (op1, op4, and op7).

### 2.7 Extraction of Metabolites from Human-Lung-Cancer Cells

A549 were seeded in 6-well Petri dishes, with each well containing 2 mL of DMEM medium (Dulbecco’s modified Eagle’s medium, Biological Industries). The cells were incubated in a 5% CO_2_ incubator at 37 °C until they reached approximately 90% confluency (2 × 105 cells). Then, 200 μL of material (op1, op4, and op7) at a concentration of 6 mg/mL was added to the medium. The cells were further incubated for 24 h. After incubation, the medium was aspirated and the cells were washed twice with 2 mL of cold phosphate-buffered saline (PBS). Subsequently, the cells were gently scraped on ice using 1 mL of PBS, collected, and centrifuged at 800*g* for 5 min. The supernatant was discarded, and 4 mL of precooled (at −80 °C) 80% (v/v) HPLC-grade methanol was added to precipitate the cells. The mixture was vortexed for 1 min and then incubated at −80 °C for 30 min. The samples were centrifuged at 4 °C and 4000*g* for 10 min, and the collected supernatant was dried using a SpeedVac (LABCONCO Refrigerated CentriVap Concentrator). The dried metabolite samples were stored at −80 °C until further analysis using mass spectrometry.

### 2.8 LC-MS-Based Targeted Metabolomics Analysis

The freeze-dried metabolite samples were reconstituted in 80 μL of a 50% acetonitrile-aqueous solution. The reconstituted samples were then centrifuged at temperatures below 4 °C at 14,000*g* for 10 min. The resulting supernatant was transferred to autosampler vials for subsequent liquid chromatography-mass spectrometry (LC-MS) analysis. To evaluate the stability and reproducibility of the analytical method, an equal volume of solution from each sample was used to prepare a quality control (QC) sample.

For the analysis, 5 μL of the reconstituted samples was injected into a 6500 QTRAP triple quadrupole mass spectrometer (SCIEX, AB SCIEX 5500) coupled with an HPLC system (Shimadzu). The metabolites were separated by using hydrophilic interaction chromatography (HILIC) on an Amide XBridge column (Waters) at a flow rate of 400 μL/min. The elution was achieved by using buffer A (20 mM ammonium hydroxide and 20 mM ammonium acetate in a water–acetonitrile mixture of 95:5 in volume) and buffer B (acetonitrile) as the mobile phases. All ions were acquired by switching between positive and negative modes with 306 selected reaction monitors. The electrospray ionization (ESI) voltage was set at +4900 V for positive mode and −4500 V for negative mode.

### 2.9 Statistical Analysis

After targeted MS/MS analysis was conducted, the molecular formula of the selected compounds was assigned. The obtained results were then imported into an Agilent Molecular Structure Correlator (MSC, B.05.00) for mass spectrometry fragmentation analysis. The structures were compared to the ion fragments displaying higher abundance, and corresponding scores were assigned. To assess the significance of differences among the metabolomics groups, a two-tailed unpaired *t*-test was employed. All statistical metabonomic analyses were performed using the MetaboAnalyst v5.0 online platform (https://www.metaboanalyst.ca/). Principal component analysis (PCA) was utilized to visualize the variations among the groups, and color ellipses representing a 95% confidence coverage were calculated based on the mean and covariance of each group’s data points. The heatmap was generated using the Ward methodology and Euclidean distance. In the volcano plots, differential metabolites with *p* < 0.1 and fold changes >2.0 were considered statistically significant. Enrichment analysis was carried out using the small molecule pathway database (SMPDB).^33^

## 3 Results and Discussion

### 3.1 Full Component Overall Analysis of High-Resolution Mass Spectrometry

**Figure 1:**
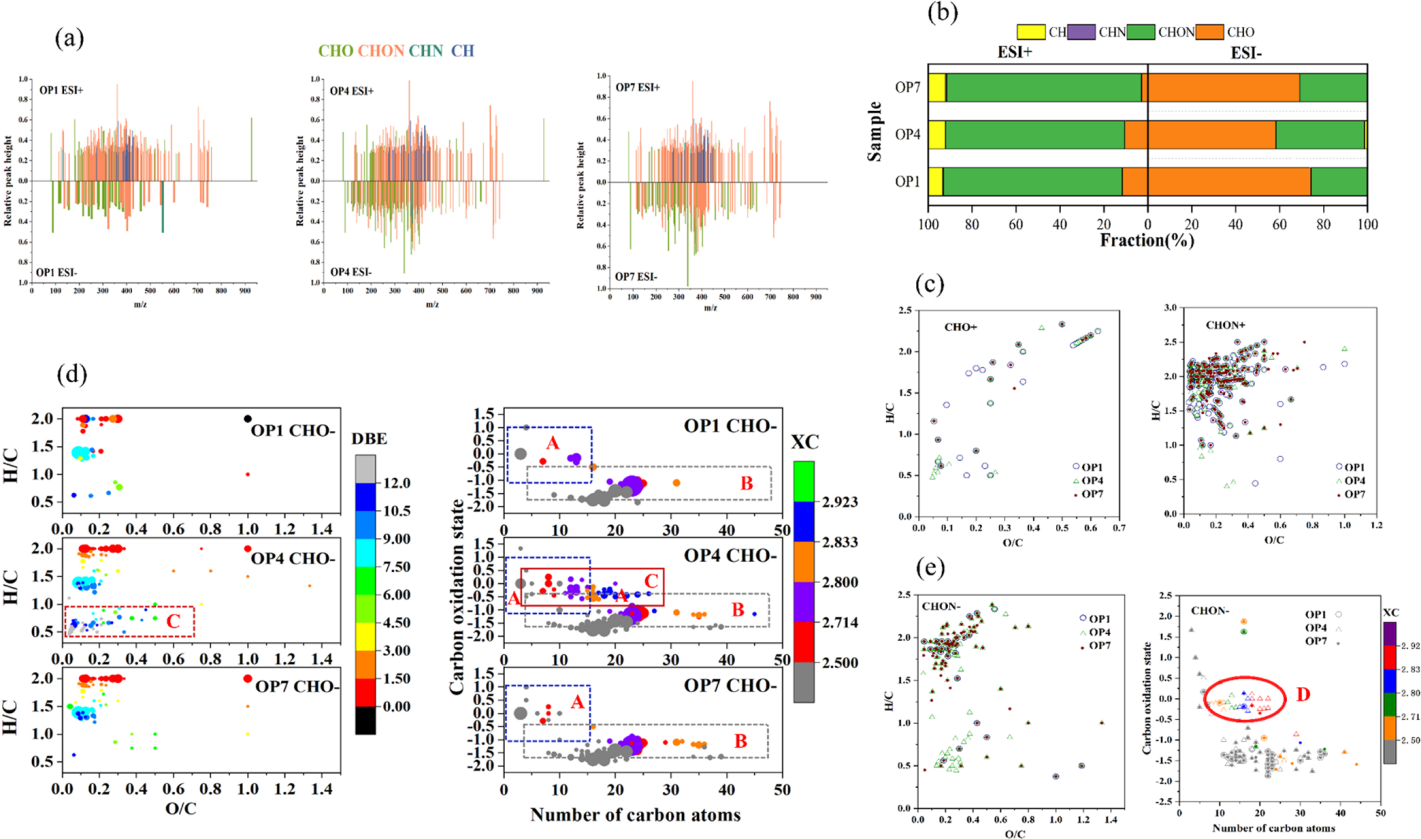
(a) Mass spectra of all compounds identified for soluble soot extracts of op1, op4, and op7 at positive and negative modes; (b) signal strength-weighted molecular fractions of all compounds identified for soluble soot particles of op1, op4, and op7 at positive and negative modes; (c) Krevelen (VK) plots of CHO and CHON compounds identified at positive mode for op1, op4, and op7 particles; (d) Van Krevelen (VK) diagrams of CHO compounds detected at negative mode, and carbon oxidation state (OS_C_) plots; and (e) Van Krevelen (VK) diagrams of CHON compounds detected at negative mode and OS_C_ plots.

Figure 1a illustrates the mass spectra of all identified compounds in soluble soot extracts obtained from op1, op4, and op7 particles using positive and negative ion modes. The peak *m*/*z* values predominantly ranged from 100 to 750 Da. In positive and negative modes, a total of 345, 312, and 310 and 73, 292, and 185 compounds were identified for the soluble soot extracts of op1, op4, and op7, respectively.

The total abundance, represented by the total peak height of chromatographic peaks, was highest for op4 particles in both positive and negative modes, approximately 3.0 × 10^7^ and 5.9 × 10^7^, respectively. This suggests that the oxidation states of soot can significantly influence the chemical properties of the particles. In negative mode, the abundance percentages of CHON and CHO soot compounds varied considerably among op1, op4, and op7. The percentage of CHON compounds increased from 25.48% in op1 to 40.28% in op4 and then decreased to 30.75% in op7. Conversely, the abundance percentage of CHO compounds decreased from 74.29% in op1 to 58.3% in op4 and then increased to 69.25% in op7. No significant differences in the abundance percentages of various compounds were observed in positive mode. The VK diagram of CHO and CHON compounds demonstrated that there were no significant differences in the positive mode regarding the chemical composition of soot for the three oxidation states, as depicted in Figure 1c. Therefore, the subsequent investigation focused on the CHON and CHO compounds identified in the soluble soot extracts of op1, op4, and op7 in negative mode to elucidate the differences in chemical composition among the soot extracts generated under the three combustion conditions.

Figure 1d presents the VK diagrams of CHO compounds detected in negative mode along with the OSc plots representing different ranges of DBE and *X*_c_. The symbol size in the plots indicates each substance’s abundance. The VK diagram reveals that the O/C values of soluble soot extracts from the three oxidation states fall within the range of 0–0.3, while the op4 samples exhibit higher O/C values, primarily between 0 and 0.6. Therefore, it can be concluded that CHO compounds for op4 are with higher degrees of unsaturation in the negative ESI mode compared to those for op1 and op7. This conclusion is further supported by the presence of unique chemical compounds in the soluble soot extracts of op4, which are not found in op1 and op7 samples, as indicated by area C in Figure 1d. These unique chemical compounds possess O/C and H/C values ranging from 0 to 0.5 and 0.4–1.0, respectively, with OSc values ranging between −0.5 and 0.5. They contain more than 6 carbon atoms but less than 27 and have *X*_c_ values >2.5. These compounds are oxygen-containing aromatic compounds, with the number of oxygen atoms varying from approximately 1 to 4. They are semivolatile or low-volatile oxidized organic components (SV-OOA and LV-OOA), most likely resulting from multistep oxidation reactions. Compounds in area B with −0.5 < OSc < −1.5 and a carbon atom count greater than 6 are categorized by previous studies as primary biomass burning organic aerosol (BBOA).^34−36^

Figure 1e presents the VK diagrams of CHON compounds identified in negative mode and the corresponding OSC plots. The analysis obtained from Figure 1a revealed that CHON compounds’ number and cumulative peak heights in the collected op1, op4, and op7 samples ranged from 45.21 to 56.76% and from 25.48 to 40.28% in negative mode, respectively. The VK diagram indicates that the O/C values of CHON compounds detected in the three samples range between 0 and 0.5, which is lower compared to that of environmental samples (0–0.7). Notably, only op4 had a higher significant number of compounds in area D, while they are nearly absent in op1 and op7. These compounds primarily contain 8–22 carbon atoms and 3–5 oxygen atoms, with an aromaticity equivalent *X*_c_ ranging from 2.5 to 2.923. Approximately 71% of the compounds (*O*/*N* ≥ 3) contain a nitro or nitroxy group. MS/MS analysis confirmed that these compounds are nitroaromatic compounds (e.g., C_8_H_7_NO_3_) with high unsaturation. Previous studies suggested that biomass burning is a source of primary nitroaromatic compounds in organic aerosols.^37^ However, this study found that for the medium oxidation state, the combustion of propane in the laboratory could also produce this type of substance.

### 3.2 Identification of Characteristic Substances

**Figure 2:**
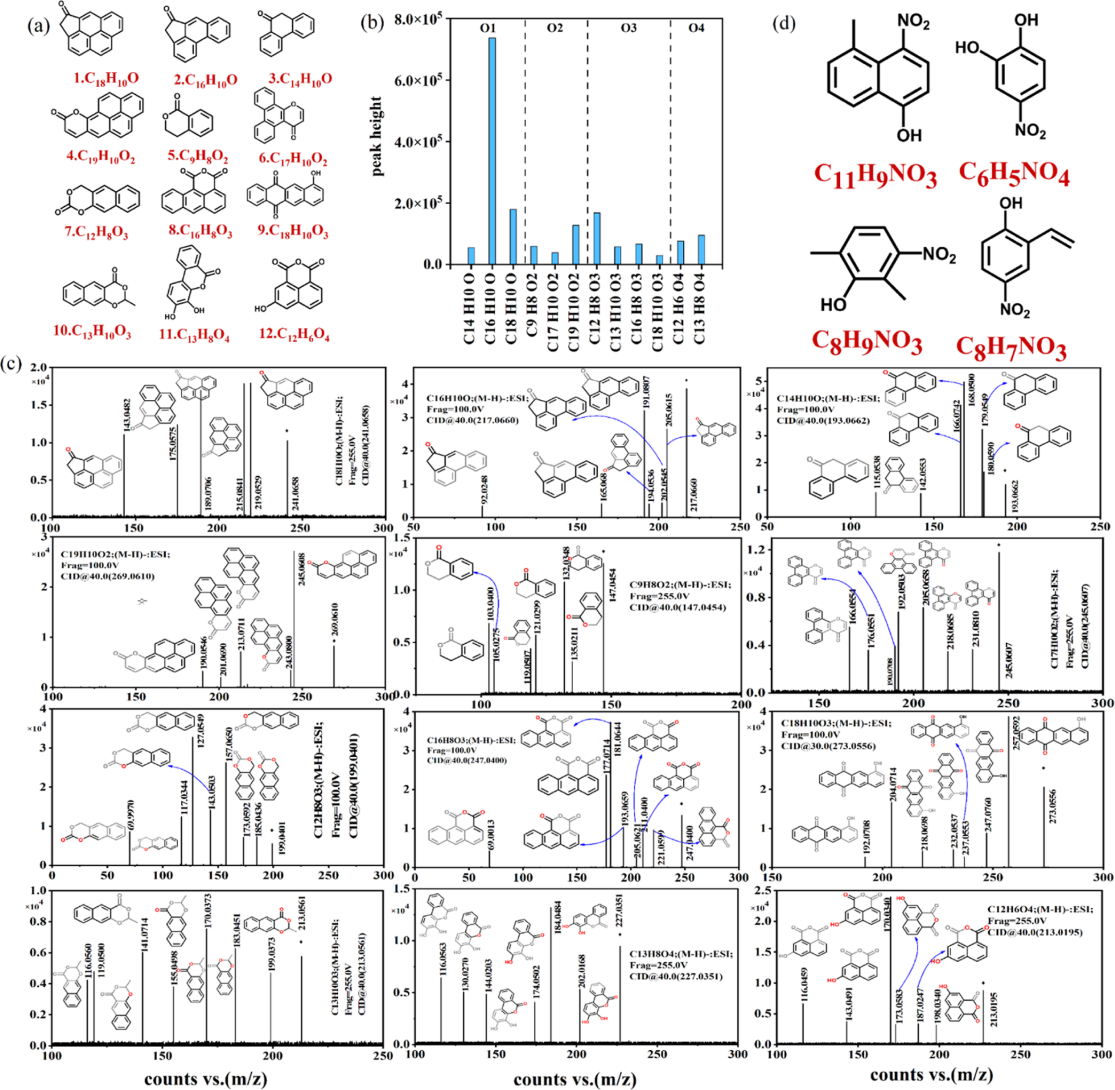
(a) Characteristic structure of CHO compounds (oxygen-containing aromatic compounds) in soluble soot particles of op4; (b) peak heights of the above characteristic compounds; (c) target MS/MS fragment ion mass spectrometry for characteristic compounds (oxygen-containing aromatic compounds) in soluble soot particles of op4; (d) characteristic (nitroaromatic compound) structure for the CHON compound in soluble soot particles of op4.

Analysis in Section 3.1 identified unique chemical compounds in soluble soot extracts of op4, characterized by the presence of at least one benzene ring (*X*_c_ > 2.5). To investigate the link between chemical structures and cellular metabolic processes at the molecular level, targeted MS/MS methodology and MSC were employed to identify the molecular structures of these compounds. A total of 128 oxygenated aromatic compounds were identified in the soluble soot extracts of op1, op4, and op7 in negative mode, with 78 species being identified in op4, accounting for approximately 61% of the total number. These compounds were associated with the compounds found in region C in Figure 1d. Figure 2a illustrates the structures of 12 oxygen-containing polycyclic aromatic hydrocarbons (OPAHs) belonging to region C for the op4 oxidation state, which were relatively more abundant among all of the identified OPAHs. The corresponding peak heights are listed in Figure 2b. The MS/MS analysis of these characteristic compounds is presented in Figure 2c. Most of these compounds consist of multiple benzene rings and unsaturated carbonyls including ketones, esters, and quinones. Previous studies by Malmborg et al. and Le et al. have also reported the production of a significant number of carbonyl compounds using miniCAST.^26,27^ And Georgios Karavalakis indicated that in comparison to pure diesel fuel, the use of biodiesel–diesel blends generally leads to reduced emissions of PAHs, nitro-PAHs, and oxygenated-PAHs.^38^ Moreover, quinones have been identified as toxic substances in secondary organic aerosols of naphthalene, attributed to their involvement in redox reactions and catalytic formation of free radicals.^39,40^ Therefore, the soluble soot extracts from op4 may exhibit stronger potential cytotoxicity in comparison to op1 and op4. The toxicity of OPAHs is closely related to oxidative stress.^41^ Exposure to phenanthrenequinone and anthracenedione promotes the expression of several oxidative stress-related genes and decreases the oxygen consumption rate of mitochondrial respiration.^41^ Furthermore, Cai et al. discovered that compounds containing carbonyl groups induce oxidative stress and promote inflammatory signaling.^42,43^

81 nitroaromatic CHON compounds have been identified in op4, constituting 61.73% of the total species present in the op4 soluble soot extract. These compounds correspond to the compounds found in area D, as depicted in Figure 1e. Figure 2d presents four potential typical structures of nitroaromatic compounds with high abundance. Nitroaromatic compounds are commonly associated with emissions from biomass burning,^37,44^ motor vehicle exhaust,^45^ or the photooxidation of aromatic VOCs in the presence of NOx,^46^ and NO_3_^•^ reaction with aromatic species.^47^ This study discovered that nitroaromatic compounds could also be generated using miniCAST under medium oxidation conditions. The toxicological implications of nitroaromatic compounds in aerosols have received limited investigation and require further exploration in future studies.^48^

### 3.3 Metabolic Profiling for A549 Cells Incubated with Soot Organics

**Figure 3:**
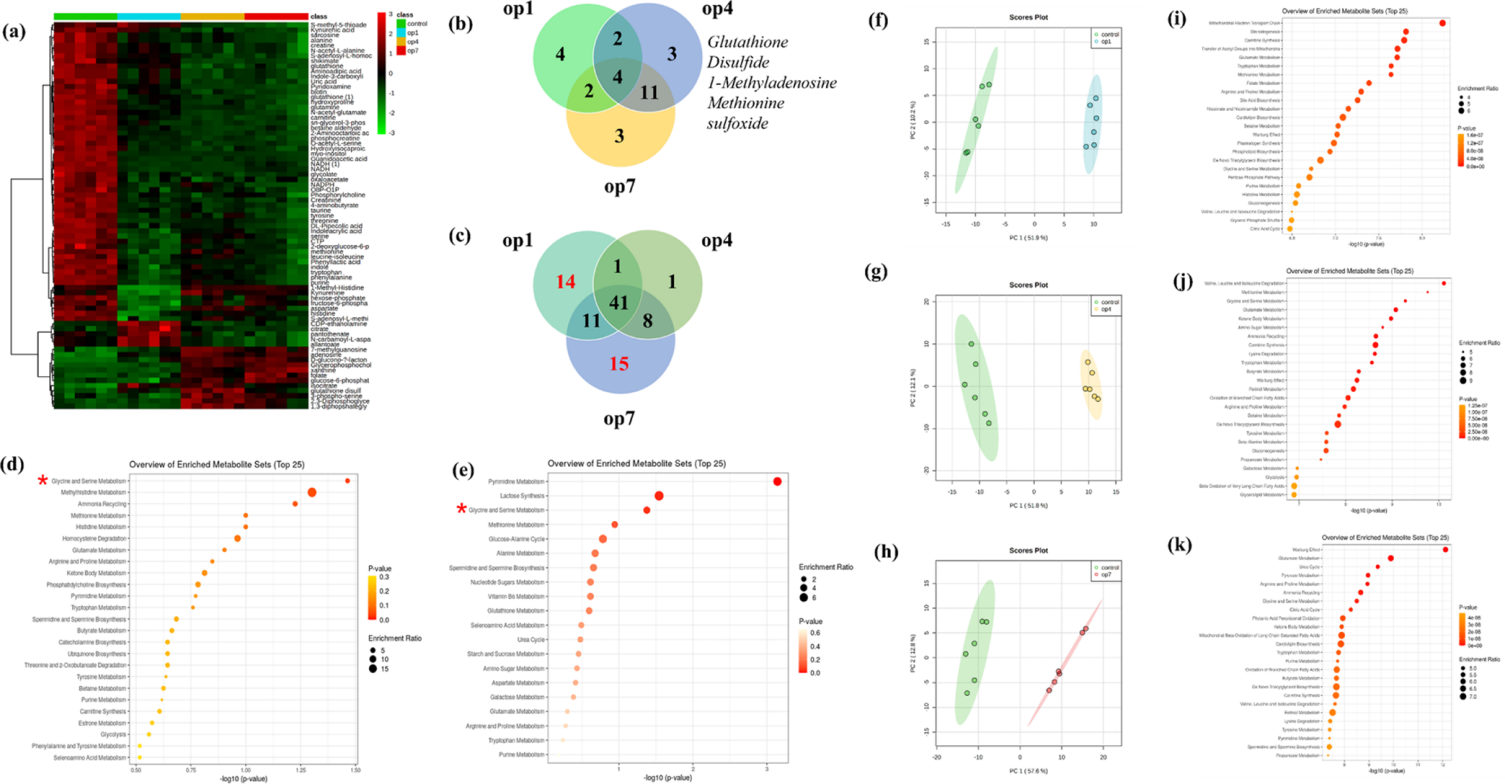
Metabolic profiles of A549 cells (control, green), op1+A549 cells (blue), op4+A549 cells (yellow), and op7+A549 cells (red). (a) Heatmap analysis of the different signature metabolites in A549 cells; red indicates the upregulation of metabolites, and green indicates the downregulation of metabolites. (b) Venn diagram of the different upregulation of metabolites from A549 cells incubated with op1, op4, and op7. (c) Venn diagram of the different downregulation of metabolites from A549 cells incubated with op1, op4, and op7. (d) Compared with the control group, regulated metabolic pathways of the downregulation of metabolites (14) in the A549 cells (op1 influence) by enriched KEGG pathway analysis. (e) Compared with the control group, regulated metabolic pathways of the downregulation of metabolites (15) in the A549 cells (op7 influence) by enriched KEGG pathway analysis. (f–h) Score plots from the PCA model for the four groups. Metabolism pathway analysis of the four groups: (i) analysis between the op1 group and control group. (j) Analysis between the op4 group and control group and (k) analysis between the op7 group and control group.

In order to investigate the biological processes triggered by the exposure of A549 cells to soluble soot extracts (op1, op4, and op7) and identify potential differential metabolites and key chemical compounds, we conducted further analysis of the measured metabolites (Supporting Information Tables S4–S6). Principal component analysis (PCA) results, illustrated in Figure 3f–h, clearly demonstrate the discrimination of A549 samples stimulated by different extracts (op1, op4, and op7) from the control group based on the identified metabolites. The PCA analysis revealed significant variations in intracellular metabolites between the different extracts. Pathway analysis of the differential metabolites unveiled specific metabolic pathways in A549 cells stimulated by op1, op4, and op7 extracts (Figure 3i–k). Specifically, for the op1 extract, the differential metabolites were primarily associated with the mitochondrial electron transport chain, steroidogenesis, carnitine synthesis, and glutamate metabolism. In the case of the op4 extract, the differential metabolites were predominantly involved in methionine metabolism, ketone body metabolism, and valine leucine and isoleucine degradation. Regarding the op7 extract, the enriched metabolic pathways were associated with the urea cycle, pyruvate metabolism, and arginine and proline metabolism. A comprehensive analysis of the differential metabolites and their relevant metabolic pathways provides valuable insights into the impact of different op extracts on cellular metabolism.

Subsequently, we comprehensively compared metabolite expression levels among the four groups. The hierarchical clustering heatmap in Figure 3a highlights 75 statistically significant differential metabolites among the groups. In comparison to the control group, op1, op4, and op7 exhibited 12, 20, and 20 significantly upregulated metabolites, respectively. Op4 led to upregulation of three specific metabolites: glutathione disulfide, 1-methyladenosine, and methionine sulfoxide. These findings support our previous observations of higher oxidation state in op4 particles and indicate their association with the oxidative stress response in exposed cells.^49−51^ Conversely, a total of 67, 51, and 75 significantly downregulated metabolites were detected in op1, op4, and op7, respectively. Upon comparison of the op1 and op7 groups with the op4 group, 14 and 15 specifically downregulated metabolites were identified, respectively. Analysis of the downregulated metabolites through the biological pathway analysis revealed common metabolic pathways between the op1 and op7 groups, emphasizing the crucial role of serine/glycine metabolism in sustaining cell survival and promoting rapid proliferation.

### 3.4 Metabolic Pathways Associated with Exposure to OPAH Standards

The HPLC-Q-TOF-MS analysis revealed that the medium oxidation state soot extracts exhibited the highest abundance of identified compounds in positive and negative modes. Further targeted MS/MS analysis distinguished two distinct groups of compounds within the medium oxidation state of soot extracts: CHO compounds, known as oxygenated polycyclic aromatic hydrocarbons (OPAHs), characterized by multiple benzene rings and unsaturated carbonyls such as ketones, esters, and quinones; and CHON compounds, specifically nitroaromatic compounds with a high degree of unsaturation. These compounds are of particular interest, as they may be associated with inducing oxidative stress in the A549 cells. The CHON compounds described above cannot be quantified by representative chemicals, so we further choose three types of OPAH standards to perform A549 cell exposure tests and metabolomics analyses, including 1, 4-naphthoquinone (C_10_H_6_O_2_) as compound A (COM-A), 9-fluorenone (C_13_H_8_O) as compound B (COM-B), and anthranone (C_14_H_10_O) as compound C (COM-C), to discuss pathways that affect metabolism by OPAHs, as given previously. These compounds can be found widely in environmental atmospheric samples, miniCAST emissions, and vehicular emissions.^52,53^

**Figure 4:**
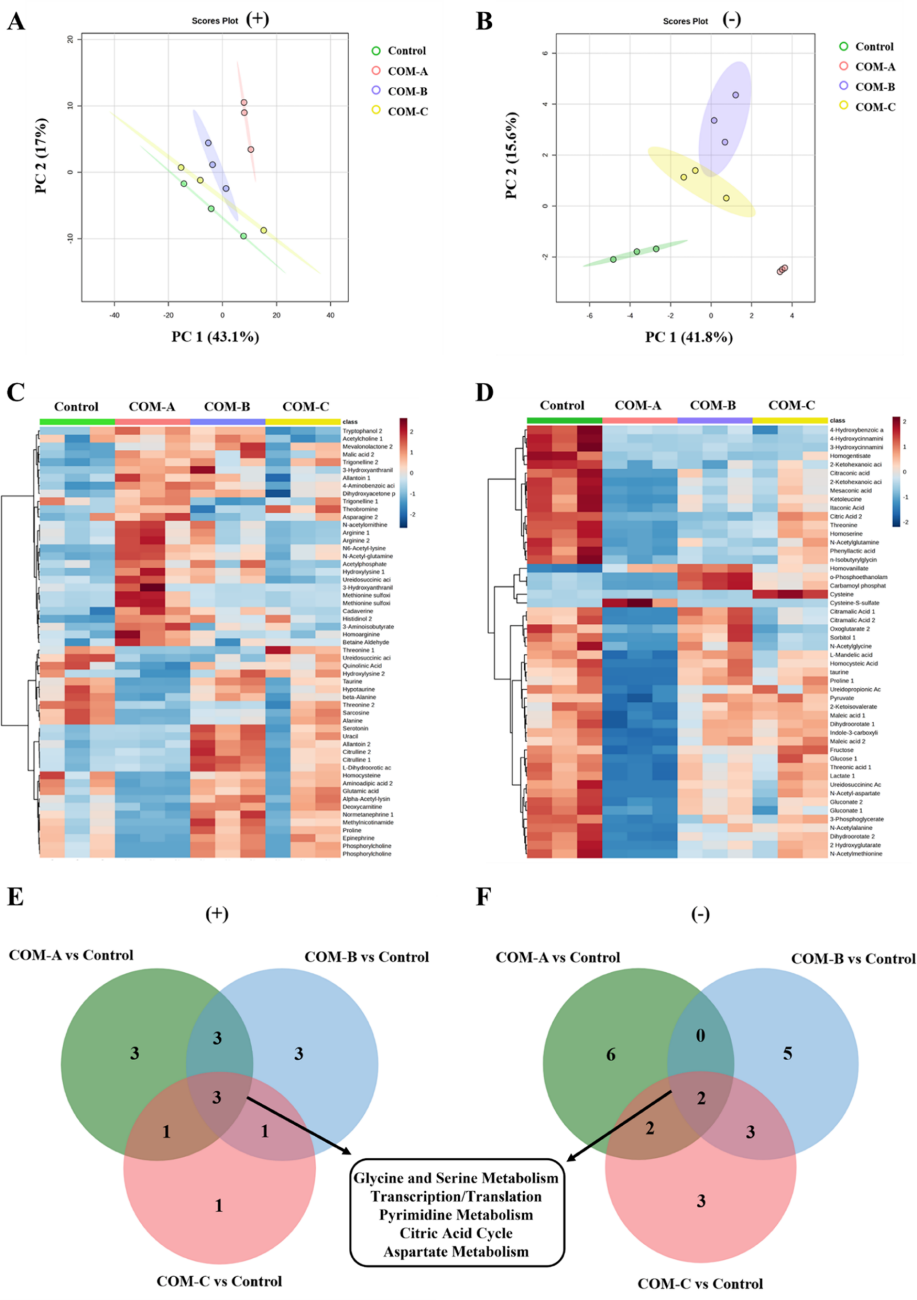
Metabolic profiles of A549 cells (control, green), COM-A+A549 cells (red), COM-B+A549 cells (blue), and COM-C+A549 cells (yellow). (A, B) Score plots from the PCA model for the four groups in positive ion mode (+) and negative ion mode (−). (C, D) Heatmap analysis of the different signature metabolites in A549 cells (positive ion mode and negative ion mode). (E, F) Compared with the control group, metabolic pathways of the downregulation of metabolites in the A549 cells by enriched KEGG pathway analysis in positive ion mode (+) and negative ion mode (−).

As shown in Figure 4a,4b, principal component analysis (PCA) can outline the original distribution of metabolites. There were no obvious outlier samples in either mode. The samples can be clearly distinguished in both ion modes with a satisfactory fit.

Hierarchical clustering heatmap of differential metabolites (VIP > 1 and *p* < 0.1) separates the cell samples in both ion modes, as shown in Figure 4c,d, respectively. Each square represents the clustering value of each metabolite in an individual sample. Red or blue color indicates increased or decreased expression of the metabolites in the samples between the two groups on the horizontal axis. Compared with the control groups, the metabolites in the A549 cells were significantly downregulated after the compound (COM-A, COM-B, and COM-C) stimulation. Integrated analysis of metabonomics in both ion modes was performed, as shown in the Venn diagram of Figure 4e,f, with downregulated metabolites overlapped in shared metabolic pathways. The downregulated pathways in the KEGG pathway analysis are shown in Figure 4e,f, including pathways in glycine and serine metabolism, transcription/translation, pyrimidine metabolism, citric acid cycle, and aspartate metabolism pathway. The obvious downregulation of these expression pathways indicates that after the stimulation of the compound, the transcription function of the cell is abnormal, and the tricarboxylic acid cycle is changed, which may affect the cell proliferation and oxidative stress response in exposed cells.^54,55^ It is noted that Figure 3 also shows downregulated pathways of serine/glycine metabolism. The results underscore the essential importance of serine/glycine metabolism in supporting cell viability and facilitating rapid cell growth.

### 3.5 PAH, OPAH, and NPAH for Vehicle Emissions with Various Fuels

**Figure 5:**
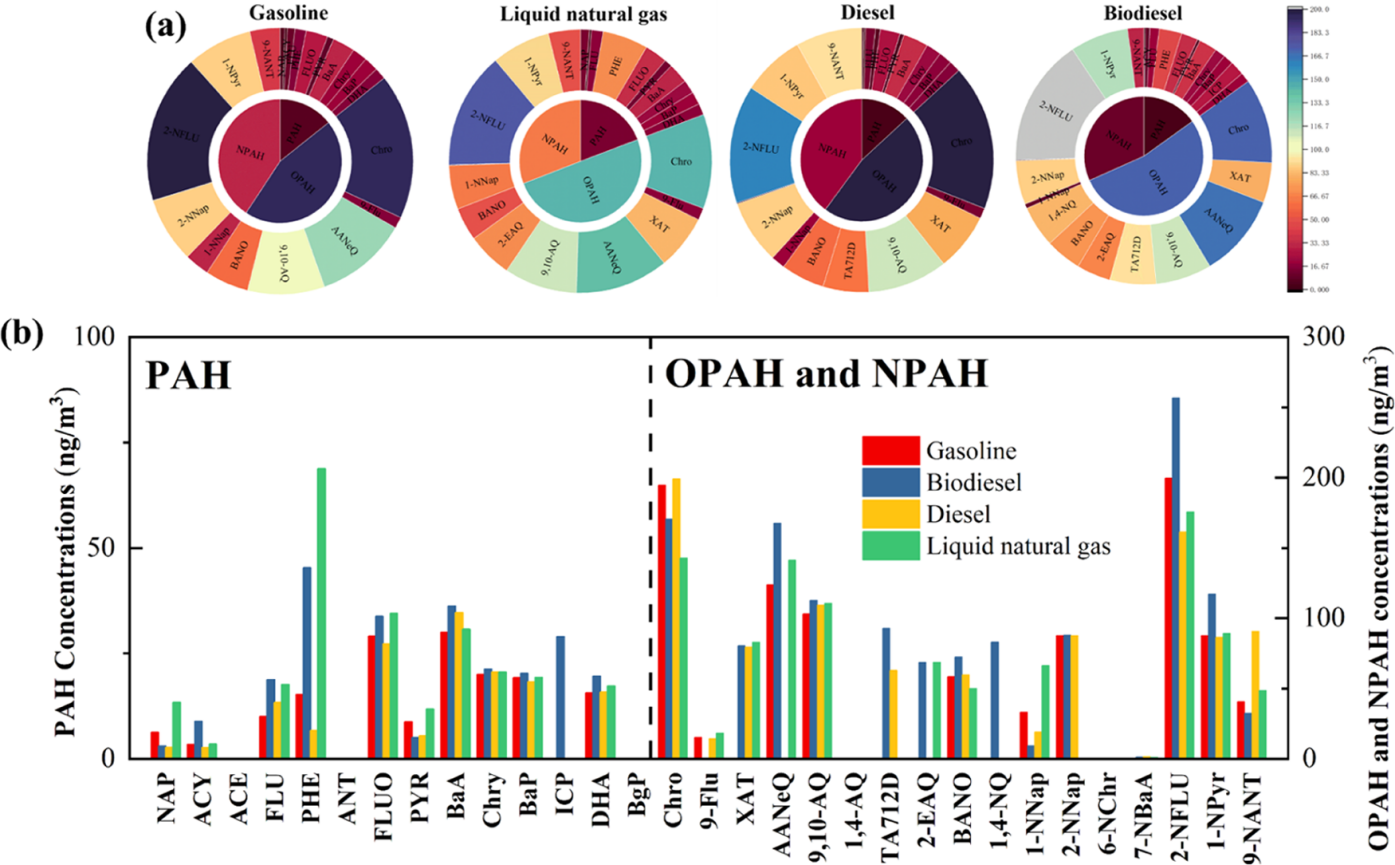
(a) Fractions and (b) concentrations of chemicals belonging to PAH, OPAH, and NPAH for vehicle emissions fueled with gasoline, biodiesel, diesel, and liquid natural gas. **OPAHs** (9Flu: 9-fluorenone, TA712D: benz[*a*]anthracene-7.12-dione, 1,4NQ: 1.4-naphthoquinone, XAT: xanthone, 2EAQ: 2-ethyl-anthraquinone, Chro: chromone, 1,4AQ: 1,4-anthraquinone); **PAHs** (NAP: naphthalene, ACY: acenaphthylene, ACE: acenaphthene, FLU: fluorene, PHE: phenanthrene, ANT: anthracene, FLUO: fluoranthene, PYR: pyrene, BaA: benz[*a*]anthracene, Chry: Chrysene, BaP: benzo[*a*]pyrene, ICP: indeno[1,2,3-cd]pyrene, DHA: dibenz[*a*,*h*]anthracene-d14, BgP: benzo[*g*,*h*,*i*]perylene); **NPAHs** (1NTN: 1-nitronaphthalene, 2NFLU: 2-nitro-9H-fluorene, 6NChr: 6-nitrochrysene, 1-NPyr: 1-nitropyrene, 9NANT: 9-nitroanthracene, 7NBaA: 7-nitrobenz(a)anthracene).

Figure 5 shows the PAH/OPAH/NPAH concentrations and fractions for the identified species produced from vehicle emissions fueled with gasoline, biodiesel, diesel, and liquid natural gas.

We identified 14 types of PAH, 10 types of OPAH, and 8 types of NPAH in total. The results indicate that the total concentrations of PAHs, OPAHs, and NPAHs follow the order: gasoline (1101.06 ng/m^3^) < diesel (1118.80 ng/m^3^) < natural gas (1232.97 ng/m^3^) < biodiesel (1594.55 ng/m^3^), and the corresponding OC concentrations were 1.37, 1.28, 1.52, and 1.22 μg C/cm^2^. It indicates that the total concentrations of PAHs, OPAHs, and NPAHs for biodiesel combustion emissions took up a relatively larger proportion to the OC components. Furthermore, PAH, OPAH, and NPAH concentrations in biodiesel emissions are still higher than those in diesel emissions. Regarding OPAH compounds, quinones and 9,10-anthraquinone are the dominant compounds, especially in diesel and gasoline exhausts (58.82% in diesel and 60.10% in gasoline). Benzoquinone shows higher emissions in the other three exhaust types but is missing from diesel emissions. 1,4-Naphthoquinone is only detected in biodiesel (9.79%). Benzanthrone is detected in both diesel and biodiesel, accounting for 10.94–11.96%. For NPAH compounds, 2-nitrofluoranthene is the predominant compound, accounting for 36.15–50.83% of the emissions. Additionally, 2-nitronaphthalene and 1-nitropyrene also exhibit relatively high emissions, except for 2-nitronaphthalene, which is not detected in natural gas emissions, with both accounting for nearly 20% of the NPAH emissions. As for PAHs, phenanthrene (4.53–29.0%), fluoranthene (14.01–18.51%), and benzo[*a*]pyrene (12.95–23.50%) show relatively high emissions. It is thought that biodiesel produces lower particulate matter (PM) levels and is considered a “green energy” alternative to diesel fuel. However, in this experiment, we discovered that biodiesel had higher levels of PAHs, OPAHs, and NPAHs compared to diesel fuel, which may have more adverse health impacts. Overall, the significant emissions of hazardous PAHs, OPAHs, and NPAHs from a wide range of traffic vehicles running on different fuels reaffirm the crucial role of the traffic source in contributing to health impacts. In this study, we identified health-hazardous organic compounds, oxygen-containing polycyclic aromatic hydrocarbons (OPAHs) consisting of fused benzene rings and unsaturated carbonyls (ketones, esters, and quinones). These compounds lead to oxidative stress. The methodology demonstrated in this study can be used to reassess the metrics used for toxicological assessments, as we strive to develop effective emission control technologies concerning fuel types and their associated biological responses.

In this study, mass spectrometry was employed to establish relationships between potentially chemical components and their impact on human alveolar epithelial cells through metabolomics-based evaluations. Targeted analysis using MS/MS revealed that particles in a moderate oxidation state exhibited the highest compound abundance, particularly oxygenated polycyclic aromatic hydrocarbons (OPAHs) consisting of condensed aromatic rings. LC-MS/MS-targeted metabolomics analysis indicated that exposure to these compounds can influence transcriptional functions, the tricarboxylic acid cycle, cell proliferation, and the oxidative stress response. Even though we quantified that biodiesel combustion exhibited higher concentrations of PAHs, OPAHs, and NPAHs, future studies should further investigate the toxicities of OPAHs originating from different emission sources.

## Supporting Information Available

Operation conditions of vehicles (2.2 S); descriptions of double bond equivalent (DBE) (2.3 S); MiniCAST emission sampling (Figure S1); sampling of vehicle emission with various fuels (Figure S2); VK plots for the repeated analysis using HPLC-Q-TOF-MS for the other two samples (Figure S3); technical parameters of sampling vehicle1 (Table S1); technical parameters of sampling vehicle2 (Table S2); technical parameters of sampling vehicle3 (Table S3); upregulated metabolites detected in samples (Table S4); downregulated metabolites detected in samples (Table S5); and metabolic pathways of apparently disturbed metabolites (Table S6) (PDF)
