# Supplementary material for: Influence of Polycyclic Aromatic Compounds and Oxidation States of Soot Organics on the Metabolome of Human-Lung Cells (A549): Implications for Vehicle Fuel Selection
Source: Environ Sci Technol. 2023 Nov 13;57(51):21593–604. doi: 10.1021/acs.est.3c05228 (PMC11441721; doi:10.1021/acs.est.3c05228)
Supplement: Supplementary file 1 — es3c05228_si_001.pdf [file es3c05228_si_001.pdf]

Supplement for

Influence of Polar Polycyclic Aromatic Compounds and Oxidation States of Soot Organics on the Metabolome of Human Lung Cells: Implications for Vehicle Fuel Selection

Lina Wang[1, 2], Wen Wen[1], Jiaqian Yan[1], Runqi Zhang[1], Chunlin Li[3], Hongxing Jiang[1], Shaofeng Chen[1], Michal Pardo[3], Ke Zhu[1], Boyue Jia[1], Wei Zhang[1], Zhe Bai[4], Longbo Shi[1], Yingjun Cheng[1, 2], Yinon Rudich[3], Lidia Morawska[5], Jianmin Chen[1, 2, 6, *]

[1]Shanghai Key Laboratory of Atmospheric Particle Pollution and Prevention (LAP[3]), Department of Environmental Science and Engineering, Fudan University, Shanghai 200438, China

[2]Shanghai Institute of Pollution Control and Ecological Security, Shanghai 200092, China

[3]Department of Earth and Planetary Sciences, Weizmann Institute of Science, Rehovot 76100, Israel

[4]School of Ecology and Environment, Inner Mongolia University, China

[5]International Laboratory for Air Quality and Health (ILAQH), School of Earth of Atmospheric Sciences, Queensland University of Technology, Brisbane, Queensland 4001, Australia

[6]IRDR International Center of Excellence on Risk Interconnectivity and Governance on Weather/Climate Extremes Impact and Public Health, Institute of Atmospheric Sciences, Fudan University, Shanghai 200438, China

***Corresponding Author**: jmchen@fudan.edu.cn

Summary: 15 pages, 3 figures, 6 tables.

2.2 S Operation conditions of vehicles
2.3 S Descriptions of double bond equivalent (DBE)
Fig. S1. MiniCAST emission sampling
Fig. S2. Sampling of vehicle emission with various fuels
Fig.S3 VK plots for the repeated analyzing using HPLC-Qtof-MS for the other two samples
Table S1 Technical parameters of sampling vehicle1
Table S2 Technical parameters of sampling vehicle2
Table S3 Technical parameters of sampling vehicle3
Table S4 Upregulated metabolites detected in samples
Table S5 Downregulated metabolites detected in samples
Table S6 Metabolic pathways of apparently disturbed metabolites

2.2 S Operation conditions vehicles

1) **Vehicle Preparation:** Prior to testing, ensure that the engine of the vehicle is in a heated state and in good mechanical condition. The engine should undergo thorough preheating, with the engine oil temperature measured at the oil dipstick hole being at least 80°C. If temperature measurement is not feasible due to vehicle design constraints, alternative methods should be employed to verify that the engine is operating within the normal temperature range. Before conducting the emission measurements, perform three free-acceleration cycles or equivalent procedures to purge the exhaust system of any residual contaminants.
2) **Sampling Procedure:** Visually inspect the vehicle's exhaust system components for any leaks. For all engines, including those with exhaust gas turbochargers, ensure that the engine is at idle at the start of each free-acceleration cycle. For heavy-duty vehicle engines, release the throttle pedal and wait for at least 10 seconds. During free-acceleration measurements, the throttle pedal must be fully depressed continuously within 1 second to maximize the fuel supply system's delivery rate in the shortest possible time. For each free-acceleration measurement, the engine must reach the cut-off speed before releasing the throttle pedal. For vehicles equipped with automatic transmissions, the engine should reach its rated speed (and if not attainable, it should not fall below 2/3 of the rated speed). During sampling, the vehicle's engine speed should be maintained at a constant level for a specific duration, simulating typical driving conditions to ensure effective sample collection. The sampling probe should extend 10 cm into the exhaust outlet to ensure that the collected samples are representative of the vehicle's exhaust

emissions. The vehicle maintains a speed of 1500-2000r/min. The sampling time period is 30 minutes. The samping air flow rate is 33L/min. For each fuel, we repeated the sampling for four times.

2.3 S In order to further characterize the chemical properties of the substance measured in the sample, the related parameters involved in the analysis process and their calculation formula are as follows. The unsaturated properties of organic compounds are usually characterized by double bond equivalent (DBE), aromatic equivalent (Xc) and aromatic index (AI). DBE provides the information of molecular ring and double bond number, and its calculation formula is shown in (1) :

$$\mathrm{DBE} = \mathrm{c} + \frac{n}{2} - \frac{h}{2} + 1 \tag{1}$$

However, when the compound contains heteroatoms (such as O, N, and S), DBE cannot accurately represent unsaturation. In order to further characterize aromatic compounds containing carbon, hydrogen, nitrogen, oxygen, sulfur and phosphorus, aromatic equivalent (Xc) is introduced.

The calculation formula of aroma index (AI) is shown in equation (3). In the formula, DBE_{AI} is the minimum number of C-C double bond addition loops in the ordinary molecular structure containing heteroatoms, and CAI is the number of carbon reduced by the number of potential double bonds contributed by heteroatoms. If DBE_{AI} or C_{AI} ≤0, AI=0.

Compared with O/C, the oxidation state (OSc) of carbon can better reflect the oxidation degree of organic compounds in the atmosphere [86], and it is widely used to describe the oxidation and aging characteristics of secondary organic aerosols. Its calculated as equation (4):

$$OS_c \approx \frac{2O}{C} - \frac{H}{C} \tag{4}$$

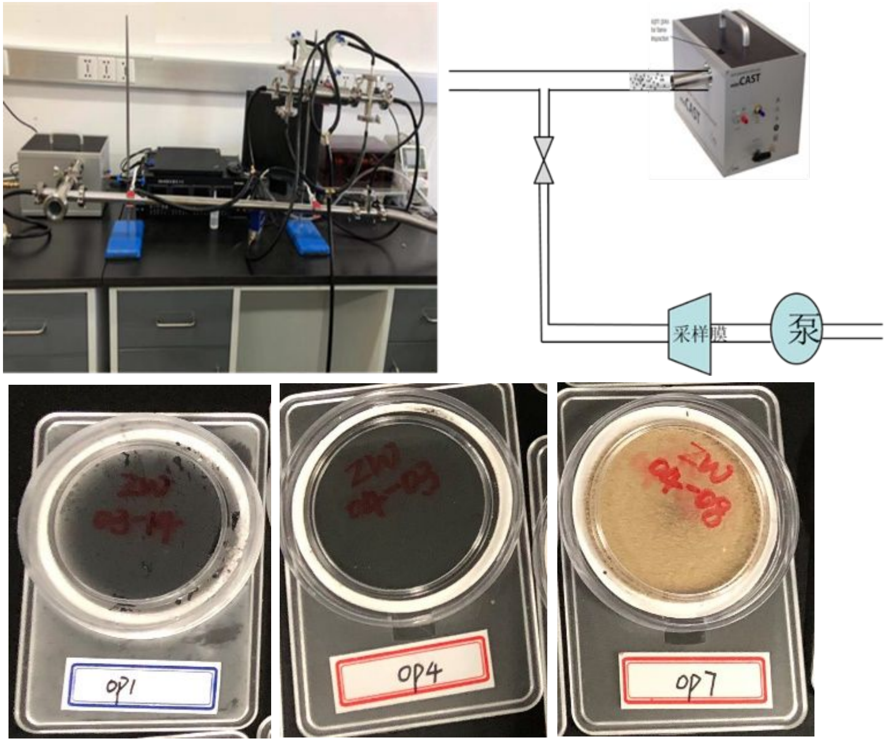

NO.	Propane (ml/min)	Oxidized air (l/min)	Fuel nitrogen mixture (l/min)	Quenched nitrogen (l/min)	Diluent air (l/min)
**op1**	60	4.0	0.0	20	10
**op4**	60	3.6	0.6	20	10
**op7**	60	2.2	0.8	20	10

Fig. S1. MiniCast emission sampling and different running conditions (op1, op4, op7)

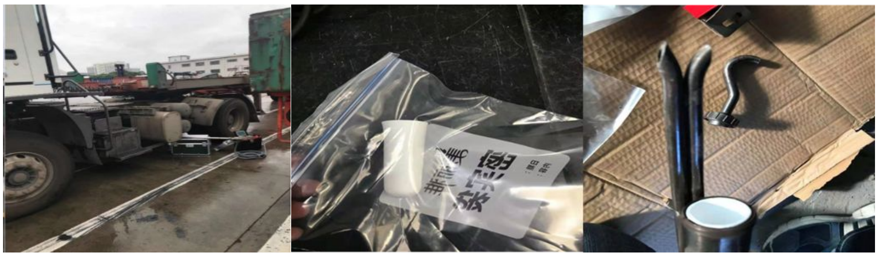

Fig. S2. Sampling of vehicle emission with various fuels

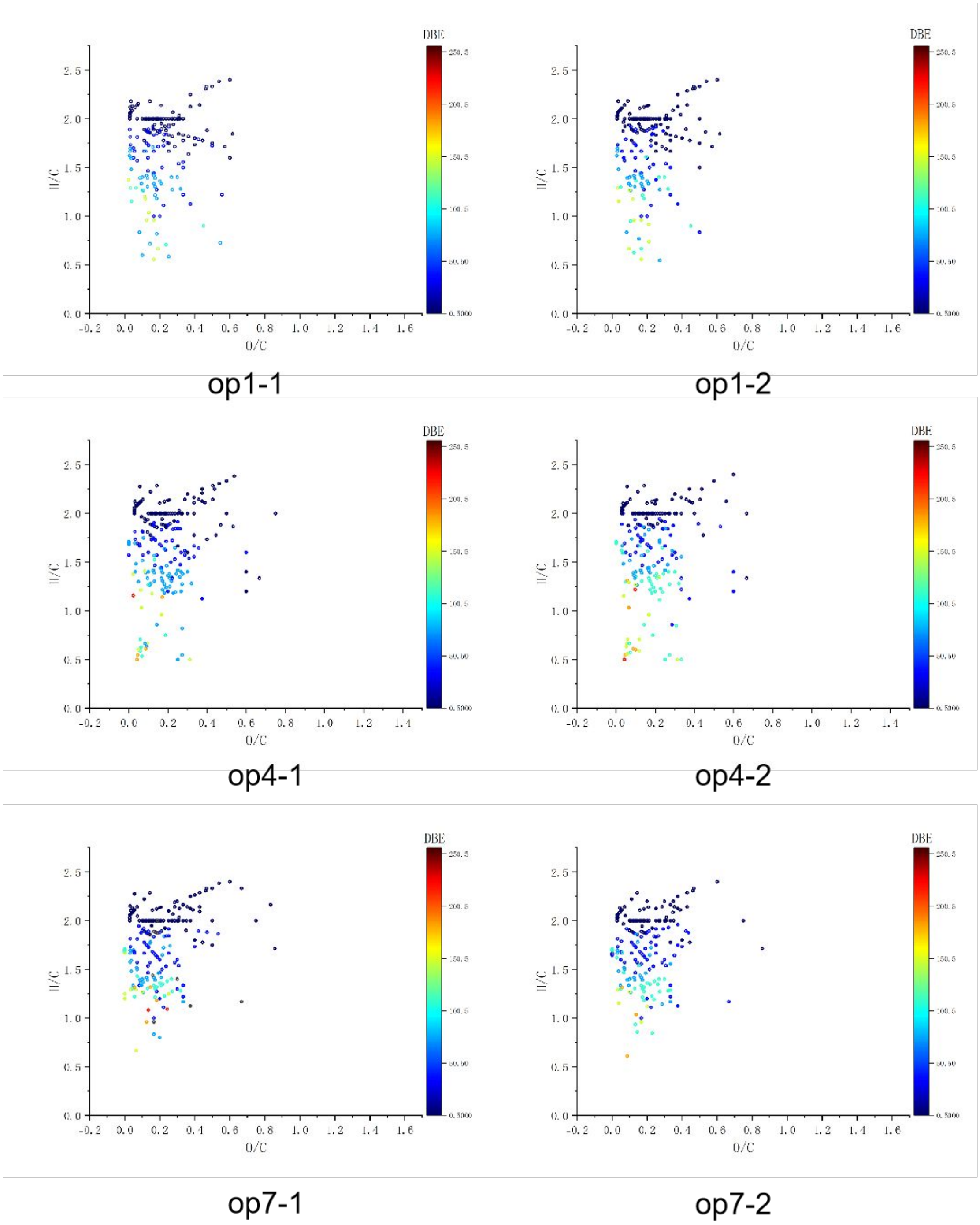

Fig. S3 VK plots for the repeated analyzing using HPLC-Qtof-MS for the other two samples

Table S1 Technical parameters of sampling vehicle1

Parameters	National V
Vehicle mass（kg）	15795
Length×width×height（mm）	10000×2550×3970
Wheel base（mm）	5800
Engine capacity（L）	4
Rated power（KW）	125
rotate speed（r/min）	2000

Table S2 Technical parameters of sampling vehicle2

Parameters	National V
Vehicle mass（kg）	4300
Engine capacity（L）	4
Rated power（KW）	173
Rotate speed（r/min）	1500-2000

Table S3 Technical parameters of sampling vehicle3

Parameters	National V
Vehicle mass（kg）	1840
Engine capacity（L）	2.4
Rated power（KW）	123
Length×width×height（mm）	5213×1847×1750

Table S4 Upregulated metabolites detected in samples

Names（up）	total	elements	formula
op1 op4 op7	6	Adenosine;	$C_{10}H_{13}N_5O_4$
		D-glucono-lactone-6-phosphate;	$C_6H_{11}O_9P_1$
		5-methyl-THF;	$C_{20}H_{25}N_7O_6$
		isocitrate;	$C_6H_8O_7$
		Deoxycholic acid;	$C_{24}H_{40}O_4$
		4-phosphopantothenate	$C_9H_{18}N_1O_8P_1$
op1 op7	2	Inosine	$C_{10}H_{12}N_4O_5$
		glucosamine	$C_6H_{13}N_1O_5$
op4 op7	18	cytidine	$C_9H_{13}N_3O_5$
		folate	$C_{19}H_{19}N_7O_6$
		xanthine	$C_5H_4N_4O_2$
		7-methylguanosine	$C_{11}H_{15}N_5O_5$
		lipoate	$C_8H_{14}O_2S_2$
		3-phospho-serine	$C_3H_8N_1O_6P_1$
		Glycerophosphocholine	$C_8H_{20}N_1O_6P_1$
		1,3-diphopshateglycerate	$C_3H_8O_{10}P_2$
		Ascorbic acid	$C_6H_8O_6$
		glutathione disulfide	$C_{20}H_{32}N_6O_{12}S_2$
		(1) retinoic acid	$C_{20}H_{28}O_2$
		2,3-Diphosphoglyceric acid	$C_3H_8O_{10}P_2$
		D-glucosamine-6-phosphate	$C_6H_{14}N_1O_8P_1$
		glutathione disulfide	$C_{20}H_{32}N_6O_{12}S_2$
		trehalose-sucrose	$C_{12}H_{22}O_{11}$
		thymidine	$C_{10}H_{14}N_2O_5$
		S-ribosyl-L-homocysteine	$C_9H_{17}N_1O_6S_1$
		(1) quinolinate	$C_7H_5N_1O_4$

op1	6	Taurodeoxycholic acid	$C_{26}H_{45}N_1O_6S_1$
		CDP-ethanolamine	$C_{11}H_{20}N_4O_{11}P_2$
		citrate	$C_6H_8O_7$
		cysteine	$C_3H_7N_1O_2S_1$
		citrate-isocitrate	$C_6H_8O_7$
		Cellobiose	$C_{12}H_{22}O_{11}$
op4	2	1-Methyladenosine	$C_{11}H_{15}N_5O_4$
		Cystine	$C_6H_{12}N_2O_4S_2$
op7	5	Uridine	$C_9H_{12}N_2O_6$
		glucose-6-phosphate	$C_6H_{13}O_9P_1$
		cholesterol	$C_{27}H_{46}O_1$
		phosphoenolpyruvate	$C_3H_5O_6P_1$
		hypoxanthine	$C_5H_4N_4O_1$

Table S5 Downregulated metabolites detected in samples

Names（down）	total	elements	formula
op1 op4 op7	4	myo-inositol	$C_6H_{12}O_6$
		NADH	$C_{21}H_{29}N_7O_{14}P_2$
		(1) NADH	$C_{21}H_{29}N_7O_{14}P_2$
		Guanidoacetic acid	$C_3H_7N_3O_2$
op4 op7	6	shikimate	$C_7H_{10}O_5$
		O-acetyl-L-serine	$C_5H_9N_1O_4$
		glycolate	$C_2H_4O_3$
		Pyridoxamine	$C_8H_{12}N_2O_2$
		2-Aminooctanoic acid	$C_8H_{17}N_1O_2$
		phenylpyruvate	$C_9H_8O_3$
op1	2	2-deoxyglucose-6-phosphate	$C_6H_{13}O_8P_1$
		1-Methyl-Histidine	$C_7H_{11}N_3O_2$
op4	3	p-hydroxybenzoate	$C_7H_6O_3$
		cholesteryl sulfate	$C_{27}H_{46}O_4S_1$
		Hydroxyisocaproic acid	$C_6H_{12}O_3$
op7	3	NADPH	$C_{21}H_{30}N7O_{17}P_3$
		phosphocreatine	$C_4H_{10}N_3O_5P_1$
		Creatinine	$C_4H_7N_3O_1$

Table S6 Metabolic pathways of apparently disturbed metabolites

Map ID	Pathways	Elements
map00250	Glutamate Metabolism	Glucosamine（up）
map00250	Glutamate Metabolism	citrate（up）
map05230	Warburg Effect	isocitrate（up）
map05230	Warburg Effect	citrate（up）
map05230	Warburg Effect	L-Cysteine（up）
map05230	Warburg Effect	phosphoenolpyruvate（up）
map00330	Arginine and Proline Metabolism	phosphocreatine（down）
map00380	Tryptophan Metabolism	quinolinate（up）
map00380	Tryptophan Metabolism	cysteine（up）
map00260	Glycine and Serine Metabolism	cysteine（up）
map00830	Retinol Metabolism	retinoic acid（up）
map00270	Methionine Metabolism	S-ribosyl-L-homocysteine（up）
map00270	Methionine Metabolism	cysteine（up）
map00270	Methionine Metabolism	Cystine（up）
map00270	Methionine Metabolism	O-acetyl-L-serine（down）
map00010	Gluconeogenesis	phosphoenolpyruvate（up）
map00760	Nicotinate and Nicotinamide Metabolism	quinolinate（up）
map00120	Bile Acid Biosynthesis	cholesterol（up）
map04913	Steroidogenesis	cholesterol（up）
map00052	Galactose Metabolism	myo-inositol（down）
map00520	Amino Sugar Metabolism	glucosamine（up）
map00410	Beta-Alanine Metabolism	quinolinate（up）
map00010	Glycolysis	phosphoenolpyruvate（up）
map00240	Pyrimidine Metabolism	cytidine（up）
map00240	Pyrimidine Metabolism	thymidine（up）
map00240	Pyrimidine Metabolism	Uridine（up）
map00620	Pyruvate Metabolism	phosphoenolpyruvate（up）
